# A Combined Ultrasonic Backscatter Parameter for Bone Status Evaluation in Neonates

**DOI:** 10.1155/2020/3187268

**Published:** 2020-05-01

**Authors:** Weiying Mao, Yang Du, Chengcheng Liu, Boyi Li, Dean Ta, Chao Chen, Rong Zhang

**Affiliations:** ^1^Department of Neonatology, Children's Hospital of Fudan University, Shanghai 201102, China; ^2^Institute of Acoustics, School of Physics Science and Engineering, Tongji University, Shanghai 200092, China; ^3^Department of Electronic Engineering, Fudan University, Shanghai 200433, China; ^4^Academy for Engineering and Technology, Fudan University, Shanghai 200433, China

## Abstract

Metabolic bone disease (MBD) is one of the major complications of prematurity. Ultrasonic backscatter technique has the potential to be a portable and noninvasive method for early diagnosis of MBD. This study firstly applied CAS to neonates, which was defined as a linear combination of the apparent integrated backscatter coefficient (AIB) and spectral centroid shift (SCS). The objective was to evaluate the feasibility of ultrasonic backscatter technique for assessing neonatal bone health using AIB, SCS, and CAS. Ultrasonic backscatter measurements at 3.5 MHz, 5.0 MHz, and 7.5 MHz were performed on a total of 505 newborns within 48 hours after birth. The values of backscatter parameters were calculated and compared among gestational age groups. Correlations between backscatter parameters, gestational age, anthropometric indices, and biochemical markers were analyzed. The optimal predicting models for CAS were determined. The results showed term infants had lower SCS and higher AIB and CAS than preterm infants. Gestational age and anthropometric indices were negatively correlated with SCS (|*r*| = 0.45 – 0.57, *P* < 0.001), and positively correlated with AIB (|*r*| = 0.36 – 0.60, *P* < 0.001) and CAS (|*r*| = 0.56 – 0.69, *P* < 0.001). Biochemical markers yielded weak or nonsignificant correlations with backscatter parameters. CAS had relatively stronger correlations with the neonatal variables than AIB and SCS. At 3.5 MHz and 5.0 MHz, only gestational age (*P* < 0.001) independently contributed to the measurements of CAS, and could explain up to 40.5% – 44.3% of CAS variation. At 7.5 MHz, the combination of gestational age (*P* < 0.001), head circumference (*P* = 0.002), and serum calcium (*P* = 0.037) explained up to 40.3% of CAS variation. This study suggested ultrasonic backscatter technique was feasible to evaluate neonatal bone status. CAS was a promising parameter to provide more information about bone health than AIB or SCS alone.

## 1. Introduction

Metabolic bone disease (MBD) is one of the major complications of prematurity, characterized by disorders of calcium and phosphorus metabolism and inadequate bone mineralization. Premature infants are at high risk of MBD because approximately 80% fetal bone mineral accretion occurs in the third trimester of gestation [1]. The lack of bone mineral deposition can be aggravated after birth by rapid bone growth, prolonged parenteral nutrition, and exposure to specific drugs such as diuretics [2]. Despite improved nutritional and medical strategies, the incidence of MBD is estimated to be 16% – 40% in infants with birth weight less than 1500 g [1]. MBD impacts both short-term and long-term prognosis of prematurity. In addition to rickets and spontaneous fractures [3], it leads to compromised lung development [3] and short stature in childhood [4]. Therefore, early diagnosis of MBD is critical.

However, it is difficult to recognize MBD with few symptoms in the early stage. Current diagnostic approaches mainly include serum biochemical markers and radiological examinations. Although the combination of serum alkaline phosphatase (AKP) > 900 IU/L and phosphorus <1.80 mmol/L yields a sensitivity of 100% and a specificity of 70% for diagnosis of low bone mineral density (BMD) [5], biochemical markers are not necessarily associated with BMD and can be affected by other diseases such as cholestasis. Repeated blood sampling is also not preferred for preterm infants. Bedside X-ray is convenient to show osteopenia and fractures, but is insensitive to a loss of bone mass less than 20% – 40% [6]. While dual energy X-ray absorptiometry (DEXA) and quantitative computed tomography (QCT) are widely used in adults to diagnose osteoporosis with high accuracy and sensitivity, they are restricted in neonates due to the risk of radiation and inconvenience to move. There is a pressing need for a valid, efficient, and noninvasive method to assess and monitor neonatal bone health.

Quantitative ultrasound (QUS) is a portable, low-cost, and radiation-free diagnostic technique which has been developed in the last decades. It not only reflects BMD [7–9], but also reflects bone microstructure [10–12] and mechanical properties [13–15]. In transmission mode, speed of sound (SOS) and bone transmission time (BTT) are commonly used parameters for neonatal bone status evaluation. Studies have shown that SOS and BTT are significantly higher in term infants than in preterm infants and positively correlated with gestational age [16–22]. On the other hand, ultrasonic transmission technique has its limitations. Scattering and dispersion are unavoidable in ultrasonic propagation, but are not considered in transmission measurements. And the requirement for a pair of transducers parallel to each other reduces the reproducibility.

Another modality of QUS, ultrasonic backscatter technique, has drawn more attention in recent years [23–29]. Unlike transmission technique, backscatter measurements are based on pulse-echo mode with a single transducer to both transmit and receive signals. It is easy to examine central skeletal sites [30, 31] and operate in incubators. Ultrasonic backscatter technique may be a promising approach to MBD screening [23–25], but few studies have been performed on neonates. Zhang et al. [32] firstly measured apparent backscatter coefficient (BSC) in 122 neonates and revealed significant correlations with gestational age, weight, and length at birth (|*r*| = 0.43 – 0.47, *P* < 0.001). Liu et al. [33] proposed a signal selection standard of apparent backscatter parameters for neonatal bone evaluation, including apparent integrated backscatter coefficient (AIB), frequency intercept of apparent backscatter (FIAB), frequency slope of apparent backscatter (FSAB), and spectral centroid shift (SCS). Our previous study [34] also suggested AIB, FIAB, and FSAB were feasible for assessing and monitoring neonatal bone status.

As different parameters reflect different properties of cancellous bone, the combination of backscatter parameters may provide more structural information. Recently, a new parameter, a linear combination of AIB and SCS (CAS), was introduced by Tang et al. [35]. They recruited 1262 adults and found that CAS was significantly correlated with BMD (|*r*| = 0.73 – 0.84, *P* < 0.05). The correlation coefficients of CAS were higher than that of AIB and SCS (|*r*| = 0.48 – 0.69, *P* < 0.05). There was no report about CAS for bone status evaluation in neonates.

We designed this study to evaluate the feasibility of ultrasonic backscatter technique for assessing neonatal bone health using AIB, SCS, and CAS. To the best of our knowledge, CAS was applied to neonates for the first time.

## 2. Materials and Methods

### 2.1. Participants

Newborns were eligible for this study who were less than 48 hours after birth and hospitalized in the Department of Neonatology, Children's Hospital of Fudan University, Shanghai, China between October 9, 2017 and May 30, 2019. Infants were excluded if born with congenital malformations, chromosomal abnormalities or inherited metabolic diseases. A total of 505 infants were enrolled, including 268 males and 237 females. All participants were divided into four groups according to gestational age: PRE-1 for preterm infants with gestational age less than 28 weeks, PRE-2 for those with gestational age between 28 weeks and 31^+6^ weeks, PRE-3 for those with gestational age between 32 weeks and 36^+6^ weeks, and TERM for term infants born at ≥37 weeks of gestation. For each infant, anthropometric indices were measured at birth including birth weight, length, and head circumference. Blood sampling was taken immediately after they were admitted to the hospital. Serum calcium, phosphorus, and AKP were tested.

Informed consents were signed by parents of each participant before enrollment. The study protocol was approved by the Ethics Committee of Children's Hospital of Fudan University (No. 25/2016).

### 2.2. Ultrasonic Backscatter Measurements

A novel ultrasonic backscatter bone diagnostic instrument (UBBD; Fudan University, Shanghai, China) was applied in this study. The backscatter signals were transmitted and received by a single planar transducer with central frequencies of 3.5 MHz, 5.0 MHz, and 7.5 MHz (Panametrics, Waltham, MA, USA) (Table 1). The transducers were excited by a bipolar short pulse with a voltage of approximately ±50 V from the UBBD instrument. Ultrasonic backscatter measurements at 3.5 MHz, 5.0 MHz, and 7.5 MHz were carried out within 48 hours after birth for each participant, and were performed with only one operator in order to avoid measurement errors caused by different operators. The transducers were placed on the medial part of the heel and coupled by ultrasonic gel (Aloka Medical Equipment, Shanghai, China), where the surface was flat and soft tissue was thin atop the calcaneus. Each measurement was finished in 5 seconds. The instrument further conducted signal preprocessing, amplification and a 14-bit analog to digital conversion with a sampling frequency of 50.0 MHz. To reduce random noise, 128 waveforms were averaged in the time domain and the signals were stored for offline analysis.

### 2.3. Ultrasonic Backscatter Parameter Calculation

**Figure 1:**
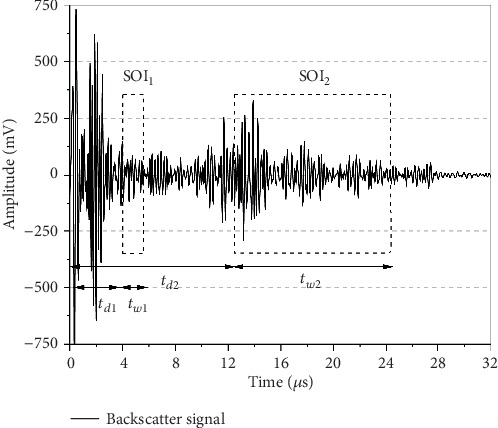
A typical ultrasonic backscatter signal from a female infant born at 29 weeks of gestation at 5.0 MHz frequency. The delay (t_d_) and duration (t_w_) of signals of interest (SOI) varies for AIB and SCS. For the SOI_1_ of AIB, t_d1_ = 4 *μ*s and t_w1_ = 2 *μ*s at 3.5 MHz, 1.4 *μ*s at 5.0 MHz, and 0.92 *μ*s at 7.5 MHz. For the SOI_2_ of SCS, t_d2_ = 12.5 *μ*s and t_w2_ = 12 *μ*s.

MATLAB R2018b (MathWorks, Natick, MA, USA) was used for signal analysis and parameter calculation.  Figure 1 illustrates a typical ultrasonic backscatter signal acquired from a female infant born at 29 weeks of gestation at 5.0 MHz. Different rectangular windows were set to select the signals of interest (SOI) for AIB and SCS. The gate delay (t_d_) of SOI_1_ for AIB was 4 *μ*s to avoided intervening signals from soft tissue and cortical bone. The duration (t_w_) was 2 *μ*s at 3.5 MHz, 1.4 *μ*s at 5.0 MHz, and 0.92 *μ*s at 7.5 MHz. The SOI_2_ for SCS located in the time range of 12.5 – 24.5 *μ*s at all the frequencies. The locations of SOIs depended on the previous study by Liu et al. [33], as well as optimization of the correlations between backscatter parameters, anthropometric indices and biochemical markers.

AIB was defined as the integrated value of apparent backscatter transfer function (ABTF) over the –6 dB frequency bandwidth [36, 37]:

(1) $$\begin{array}{c} ABTF=10\log_{10}\left(\frac{P_{s}\left(f\right)}{P_{r}\left(f\right)}\right), \end{array}$$

(2) $$\begin{array}{c} AIB=\frac{\int_{f_{\min}}^{f_{\max}}ABTF\text{ d}f}{f_{\max}-f_{\min}}. \end{array}$$

SCS was the downshift of the spectral centroid of the backscatter signal (*f*_s_) compared to that of the reference signal (*f*_r_). It was calculated as [38, 39].

(3) $$\begin{array}{c} SCS=f_{s}-f_{r}=\frac{\int_{f_{\min}}^{f_{\max}}f\cdot P_{s}\left(f\right)df}{\int_{f_{\min}}^{f_{\max}}P_{s}\left(f\right)df}-\frac{\int_{f_{\min}}^{f_{\max}}f\cdot P_{r}\left(f\right)df}{\int_{f_{\min}}^{f_{\max}}P_{r}\left(f\right)df}. \end{array}$$

In the formulas above, *f*_max_ and *f*_min_ were the upper and lower limit of the –6 dB frequency bandwidth of the transducer; P_s_(*f*) and P_r_(*f*) referred to the power spectrum of the backscatter signal and the reference signal, respectively. The reference signal was a reflected signal from a polished steel plate immersed in pure water. The power spectrum was obtained through a fast Fourier transform.

CAS was a linear combination of AIB and SCS, which was defined as [35].

(4) $$\begin{array}{c} CAS=\omega AIB-SCS. \end{array}$$

According to Tang et al. [35], the coefficient *ω* was a positive number depended on when the correlation between CAS and BMD achieved best. Considering that BMD of the newborns was unable to measure directly and fetal bone matured with increased gestational age, the value of *ω* in this study was determined by optimization of the correlation between CAS and gestational age instead. The values of *ω* were 0.041 at 3.5 MHz, 0.030 at 5.0 MHz, and 0.072 at 7.5 MHz.

For each participant, AIB, SCS and CAS were calculated at all the frequencies of 3.5 MHz, 5.0 MHz and 7.5 MHz. Different frequency bands were put into the same formula for each parameter.

### 2.4. Statistical Analyses

We used SPSS 22.0 (IBM, Armonk, NY, USA) for statistical analysis. The normality of all the variables was checked by the Shapiro-Wilk test. None obeyed normal distribution. Descriptive data were presented as median and quartile. Differences among subgroups based on gestational age were examined using the Kruskal-Wallis *H* test followed by all pairwise comparisons. Correlations between ultrasonic backscatter parameters, gestational age, anthropometric indices, and biochemical markers were calculated by simple linear regression (Spearman's rank correlation). Relative contributions of gestational age, anthropometric indices, and biochemical markers to the measurements of CAS were determined using multiple linear regression. The optimal models for predicting CAS were produced by forward stepwise multiple regression. A *P* value less than 0.05 indicated statistical significance.

## 3. Results

Table 2 summarizes the baseline characteristics of the participants. No significant difference in gender was found across subgroups. Birth weight, length, head circumference, and serum calcium of the enrolled infants increased with gestational age. Serum phosphorus and AKP at birth decreased with gestational age.

As Table 3 demonstrates, AIB and CAS were significantly higher and SCS was significantly lower in term infants compared with any group of preterm infants at 3.5 MHz, 5.0 MHz, and 7.5 MHz. That was also shown in PRE-3 when compared with either PRE-1 or PRE-2. There was no significant difference in the backscatter parameters between PRE-1 and PRE-2 except for CAS at 7.5 MHz.

**Figure 2:**
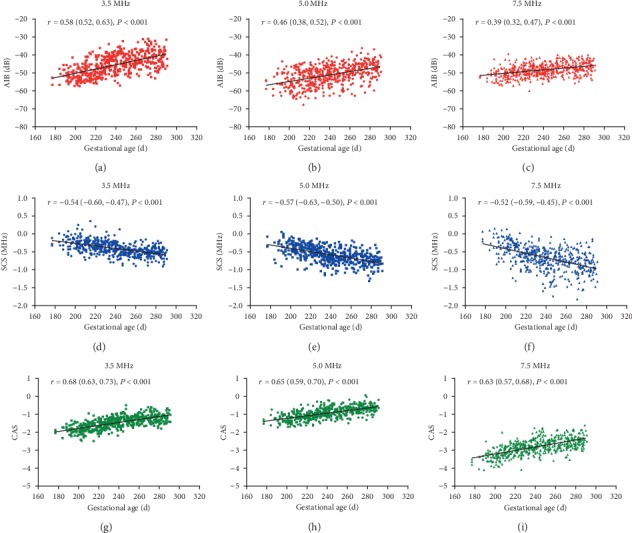
The scatterplots of AIB (a-c), SCS (d-f), and CAS (g-i) associated with gestational age at 3.5 MHz, 5.0 MHz, and 7.5 MHz (n = 505). Gestational age is negatively correlated with SCS (|*r*| = 0.52 – 0.57, *P* < 0.001), and positively correlated with AIB (|*r*| = 0.39 – 0.58, *P* < 0.001) and CAS (|*r*| = 0.63 – 0.68, *P* < 0.001).

Figure 2 is the scatterplot of AIB, SCS, and CAS associated with gestational age at 3.5 MHz, 5.0 MHz, and 7.5 MHz.  Table 4 lists the correlation coefficients of the backscatter parameters with anthropometric indices and biochemical markers at all the frequencies.

Gestational age, birth weight, length, and head circumference were negatively correlated with SCS (|*r*| = 0.45 – 0.57, *P* < 0.001), and positively correlated with AIB (|*r*| = 0.36 – 0.60, *P* < 0.001) and CAS (|*r*| = 0.56 – 0.69, *P* < 0.001). Biochemical markers, especially serum phosphorus, yielded relatively weak correlations with backscatter parameters (|*r*| = 0.18 – 0.26 for AKP, *P* < 0.001; |*r*| = 0.17 – 0.34 for calcium, *P* < 0.001; |*r*| = 0.06 – 0.14 for phosphorus, *P* < 0.05 or not significant). In most cases, CAS had stronger correlations with the neonatal variables than AIB and SCS.

Table 5 shows the correlations between gestational age, anthropometric indices, and biochemical markers. Gestational age and anthropometric indices had strong positive correlations with each other (|*r*| = 0.86 – 0.96, *P* < 0.001) and weak to moderate correlations with biochemical markers (|*r*| = 0.17 – 0.43, *P* < 0.001). Correlations among biochemical markers were quite weak or nonsignificant.

To find the variables independently influencing CAS, gestational age, anthropometric indices, and biochemical markers were included in multiple regression analysis, as shown in Table 6. At 3.5 MHz, gestational age was the only variable that significantly contributed to the measurements of CAS (*P* < 0.001). At 5.0 MHz, serum calcium (*P* = 0.040) also made an independent contribution to CAS measurements besides gestational age (*P* < 0.001). However, forward stepwise regression revealed that only gestational age was entered into the optimal model for predicting CAS at both 3.5 MHz and 5.0 MHz (Table 7). It could explain up to 44.3% and 40.5% of the variation of CAS in neonates, respectively. At 7.5 MHz, gestational age (*P* < 0.001), head circumference (*P* = 0.002), and serum calcium (*P* = 0.037) were independent factors that influenced CAS measurements, and the combination could explain up to 40.3% of the variation.

## 4. Discussion

### 4.1. Explanation of Ultrasonic Backscatter Parameters

AIB is “apparent” backscatter parameter which represents frequency-averaged backscatter power without compensation for attenuation in ultrasonic propagation [25, 27, 36, 37, 40]. AIB is convenient for in vivo measurements at a lower cost as it is unnecessary to measure the attenuation coefficient at the investigated position using transmission technique with two transducers. The values of AIB depend primarily on the comprehensive effects of backscatter and attenuation [36, 37, 41]. Attenuation is determined by the attenuation coefficient, as well as ultrasonic propagation length presented as t_d_ and t_w_ in this study. When propagation length is small, attenuation is weak and backscatter dominates the observed effects. Consequently, we selected short t_d_ and t_w_ for AIB. AIB is expected to be positively correlated with BMD in this case as the backscatter effects are more pronounced with higher BMD [33, 36, 37].

SCS is a downshift in the center frequency of the backscattered spectrum caused by attenuation within a scattering medium [42]. Stronger attenuation leads to larger magnitude of the downshift. Since the attenuation coefficient increases with BMD, the correlation between SCS and BMD is consistently negative [31, 38, 39, 42]. Relatively long t_d_ and t_w_ are preferred in order to improve the sensitivity to detect low BMD [33].

CAS is a linear combination of AIB and SCS, varying in the same direction as AIB according to the formula. As backscatter is the dominant effect that influences AIB while attenuation mainly affects SCS, CAS reflects both backscatter and attenuation effects of cancellous bone. It may be a promising backscatter parameter to provide more information about bone status than AIB or SCS alone.

### 4.2. Correlations with Gestational Age

A large number of studies on bone specimens from adults or animals have confirmed AIB and SCS were not only significantly correlated with BMD obtained from DEXA or QCT [26, 27, 31, 38, 39, 42–44], but also provided complementary information about bone microstructure and mechanical properties such as bone volume fraction, trabecular separation, thickness, and number density [23, 25, 26, 45, 46]. In the present study, newborns with older gestational age had lower SCS and higher AIB and CAS at birth, suggesting that term infants had better bone status than preterm infants. Moderate correlations were found between gestational age and the backscatter parameters which were also shown in previous studies about neonates [32–34, 47]. There was a decreasing trend in the correlation coefficients between AIB and gestational age at higher frequencies which was likely attributed to heavy attenuation [36, 37, 41], but the frequency-dependent variation of correlation coefficients was not obvious for SCS and CAS. The correlation coefficients of CAS were higher than that of AIB and SCS at all the frequencies, similar to the results of Tang and his colleagues [35]. It might be concluded that AIB, SCS and CAS were feasible to evaluate neonatal bone health at birth. CAS was probably more effective than AIB or SCS alone. Note that both BMD and bone mineral content (BMC) of newborns increased with gestational age [6], so it was reasonable to treat gestational age as an index of the degree of bone maturity.

### 4.3. Correlations with Anthropometric Indices

Anthropometric indices (birth weight, length and head circumference) were closely related to gestational age, reflecting fetal growth and maturation as well. Similarly, anthropometric indices had moderate correlations with AIB, SCS, and CAS and were able to reflect skeletal development. In accordance with gestational age, the correlation coefficients between AIB and anthropometric indices tended to decrease at higher frequencies though without significance. CAS had relatively stronger correlations with anthropometric indices than AIB and SCS at 3.5 MHz, 5.0 MHz, and 7.5 MHz. Among these anthropometric indices, head circumference was generally considered an index of neurodevelopment which was different from birth weight and length. However, Akcakus et al. [48] have reported positive correlations between head circumference and whole-body BMD and BMC of term infants at birth. Studies on ultrasonic backscatter technique also found that head circumference was significantly correlated with SCS, AIB, FIAB, and FSAB [33, 34].

### 4.4. Correlations with Biochemical Markers

Although serum calcium, phosphorus, and AKP were moderately correlated with gestational age, they were weakly or nonsignificantly correlated with ultrasonic backscatter parameters, consistent with Liu et al. [47] and our previous study [34]. Serum calcium might not be a useful marker for inadequate bone mineralization because its level usually remained normal in the early stage of MBD as a result of secondary hyperparathyroidism [49]. In contrast, the diagnostic power of serum phosphorus and AKP is still controversial. Some studies revealed that low phosphorus levels in combination with high AKP levels increased the sensitivity and specificity of MBD screening [5, 6], but there were also studies that reported routine measurements of serum AKP and phosphorus were useless in predicting bone mineralization outcome in premature infants [50]. The validity of biochemical markers for assessing neonatal bone health required further researches.

### 4.5. Optimal Predicting Models for CAS

Simple linear regression demonstrated that gestational age, anthropometric indices, and biochemical markers were significantly associated with each other. Multiple regression demonstrated only gestational age independently contributed to the measurements of CAS at all the frequencies. As discussed above, gestational age increased with the degree of fetal maturity and positively correlated with BMD and BMC of newborns, so it was not surprising that gestational age played an important role in the predicting models of CAS. Anthropometric indices and biochemical markers were not independent factors influencing CAS mainly on account of multicollinearity. It was noteworthy that head circumference and serum calcium at 7.5 MHz were also entered into the predicting model. Ultrasound with higher frequencies (i.e., 7.5 MHz) provided a better resolution for the measurement of bone microstructure. Considering the tiny bone size of premature infants, the model at 7.5 MHz was supposed to achieve better performance in predicting backscatter properties. However, the optimal model at 7.5 MHz explained up to 40.3% of the variation of CAS, slightly lower than that of 3.5 MHz and 5.0 MHz. Therefore, the optimal frequency range for neonates and corresponding mechanisms deserved more attention in the future.

## 5. Limitations

One potential limitation of this study was the lack of direct indicators of bone status for neonates. None of the anthropometric indices or biochemical markers was completely reliable substitute for BMD and microstructural parameters. If there were comparative data that directly reflected bone status, the results would be more convincing. Moreover, follow-up studies remained to be conducted in view that MBD typically arose within 6 – 16 weeks after birth [1].

## 6. Conclusions

We performed ultrasonic backscatter measurements on 505 newborns at birth at 3.5 MHz, 5.0 MHz, and 7.5 MHz frequencies. The CAS, which was defined as a linear combination of AIB and SCS, was applied to neonates for the first time. Results indicated that AIB, SCS and CAS were significantly correlated with gestational age and anthropometric indices. CAS had relatively stronger correlations than AIB or SCS alone. Gestational age made significantly independent contributions to CAS at all the frequencies, and the optimal predicting models could explain up to 40.3% – 44.3% of the variation of CAS. This study suggested ultrasonic backscatter technique was feasible to evaluate neonatal bone status. CAS was a promising parameter to provide more information about bone health.

## Figures and Tables

**Table 1:** Characteristics of the transducers used in this study.

Model	Diameter (inch)	Central frequency (MHz)	–6 dB bandwidth (band range) (MHz)
V546	0.250	3.5	3.25 (1.60 – 4.91)
V543	0.250	5.0	3.91 (3.20 – 7.11)
V122	0.375	7.5	5.65 (4.79 – 10.44)

**Table 2:** Baseline characteristics of the participants at birth.

Gestational age group	PRE-1	PRE-2	PRE-3	TERM	Total
	< 28 weeks	28 – 31^+6^ weeks	32 – 36^+6^ weeks	≥ 37weeks	
Number	25	139	195	146	505
Male	14 (56.0)	70 (50.4)	109 (55.9)	75 (51.4)	268 (53.1)
Gestational age (d)	191 (187, 193)	211 (204, 219)	240 (232, 248)	274 (266, 283)	239 (218, 263)
Birth weight (g)	990 (933, 1115)	1440 (1245, 1625)	2000 (1800, 2290)	3305 (2954, 3653)	1980 (1533, 2830)
Length (cm)	35 (34, 36)	38 (37, 40)	42 (41, 44)	49 (47, 50)	42 (39, 46)
Head circumference (cm)	25.0 (24.0, 25.0)	27.5 (26.0, 28.0)	30.0 (29.0, 31.0)	34.0 (33.0, 35.0)	30.0 (28.0, 33.0)
Alkaline phosphatase (IU/L)	261 (207, 317)	226 (173, 273)	204 (168, 238)	167 (139, 199)	197 (160, 246)
Calcium (mmol/L)	1.81 (1.66, 1.93)	1.98 (1.87, 2.12)	2.10 (1.99, 2.22)	2.17 (2.01, 2.29)	2.07 (1.94, 2.21)
Phosphorus (mmol/L)	2.06 (1.91, 2.24)	1.99 (1.78, 2.27)	1.86 (1.67, 2.03)	1.79 (1.62, 1.94)	1.88 (1.67, 2.09)

Data are n (%) or median (P_25_, P_75_).

**Table 3:** The values of ultrasonic backscatter parameters among different gestational age groups.

Gestational age group	PRE-1	PRE-2	PRE-3	TERM
	< 28 weeks	28 – 31^+6^ weeks	32 – 36^+6^ weeks	≥ 37 weeks
AIB (dB)				
3.5 MHz	–51.64 (–54.36, –48.22)	–50.71 (–53.62, –46.78)	–44.54 (–48.42, –40.65)^ab^	–41.94 (–44.95, –37.94)^abc^
5.0 MHz	–55.78 (–58.61, –50.29)	–54.01 (–58.00, –49.56)	–50.84 (–54.22, –47.74)^ab^	–47.28 (–50.49, –44.85)^abc^
7.5 MHz	–51.29 (–53.66, –49.08)	–49.60 (–51.82, –47.85)	–48.28 (–50.27, –46.09)^ab^	–46.74 (–48.62, –43.98)^abc^
SCS (MHz)				
3.5 MHz	–0.24 (–0.35, –0.10)	–0.28 (–0.40, –0.19)	–0.43 (–0.51, –0.31)^ab^	–0.51 (–0.61, –0.42)^abc^
5.0 MHz	–0.35 (–0.50, –0.14)	–0.41 (–0.53, –0.29)	–0.63 (–0.74, –0.49)^ab^	–0.72 (–0.83, –0.59)^abc^
7.5 MHz	–0.26 (–0.43, –0.09)	–0.43 (–0.65, –0.29)	–0.67 (–0.85, –0.50)^ab^	–0.81 (–1.04, –0.63)^abc^
CAS				
3.5 MHz	–1.87 (–2.01, –1.71)	–1.76 (–1.93, –1.54)	–1.38 (–1.57, –1.20)^ab^	–1.17 (–1.38, –1.03)^abc^
5.0 MHz	–1.25 (–1.50, –1.10)	–1.16 (–1.36, –1.00)	–0.91 (–1.09, –0.71)^ab^	–0.68 (–0.86, –0.56)^abc^
7.5 MHz	–3.37 (–3.61, –3.18)	–3.12 (–3.34, –2.85)^a^	–2.78 (–2.96, –2.53)^ab^	–2.51 (–2.77, –2.31)^abc^

^a^ ^b^ ^c^Data are median (P_25_, P_75_). Significantly different from PRE-1 (*P* < 0.001). Significantly different from PRE-2 (*P* < 0.001). Significantly different from PRE-3 (*P* < 0.001).

**Table 4:** Correlation coefficients of backscatter parameters with anthropometric indices and biochemical markers.

	AIB			SCS			CAS		
	3.5 MHz	5.0 MHz	7.5 MHz	3.5 MHz	5.0 MHz	7.5 MHz	3.5 MHz	5.0 MHz	7.5 MHz
Birth weight									
Spearman *r*	0.60	0.47	0.39	–0.54	–0.56	–0.51	0.69	0.65	0.62
*P* value	< 0.001	< 0.001	< 0.001	< 0.001	< 0.001	< 0.001	< 0.001	< 0.001	< 0.001
Length									
Spearman *r*	0.53	0.42	0.36	–0.52	–0.51	–0.45	0.63	0.59	0.56
*P* value	< 0.001	< 0.001	< 0.001	< 0.001	< 0.001	< 0.001	< 0.001	< 0.001	< 0.001
Head circumference									
Spearman *r*	0.57	0.44	0.39	–0.54	–0.53	–0.50	0.67	0.62	0.60
*P* value	< 0.001	< 0.001	< 0.001	< 0.001	< 0.001	< 0.001	< 0.001	< 0.001	< 0.001
Alkaline phosphatase									
Spearman *r*	–0.23	–0.20	–0.20	0.18	0.21	0.18	–0.25	–0.26	–0.26
*P* value	< 0.001	< 0.001	< 0.001	< 0.001	< 0.001	< 0.001	< 0.001	< 0.001	< 0.001
Calcium									
Spearman *r*	0.31	0.28	0.17	–0.24	–0.28	–0.30	0.33	0.34	0.32
*P* value	< 0.001	< 0.001	< 0.001	< 0.001	< 0.001	< 0.001	< 0.001	< 0.001	< 0.001
Phosphorus									
Spearman *r*	–0.11	–0.09	–0.14	0.09	0.13	0.06	–0.12	–0.14	–0.14
*P* value	0.017	0.056	0.002	0.054	0.004	0.217	0.008	0.001	0.002

**Table 5:** Correlations between gestational age, anthropometric indices, and biochemical markers.

Variables	Spearman *r*						
	Gestational age	Birth weight	Length	Head circumference	Alkaline phosphatase	Calcium	Phosphorus
Gestational age	1	—	—	—	—	—	—
Birth weight	0.90^a^	1	—	—	—	—	—
Length	0.86^a^	0.93^a^	1	—	—	—	—
Head circumference	0.91^a^	0.96^a^	0.91^a^	1	—	—	—
Alkaline phosphatase	–0.38^a^	–0.29^a^	–0.28^a^	–0.28^a^	1	—	—
Calcium	0.43^a^	0.39^a^	0.35^a^	0.38^a^	–0.12^b^	1	—
Phosphorus	–0.30^a^	–0.17^a^	–0.19^a^	–0.21^a^	0.13^b^	–0.04^c^	1

^a^ *P* < 0.001;^b^*P* < 0.01; ^c^ not significant, *P* = 0.338.

**Table 6:** Multivariate analysis of gestational age, anthropometric indices, and biochemical markers for the measurement of CAS.

Variables	3.5 MHz		5.0 MHz		7.5 MHz	
	Regression coefficient	*P* value	Regression coefficient	*P* value	Regression coefficient	*P* value
Gestational age (d)	0.006	< 0.001	0.005	< 0.001	0.008	< 0.001
Birth weight (g)	0.00006	0.191	0.0001	0.100	–0.00001	0.813
Length (cm)	0.001	0.825	–0.002	0.751	–0.013	0.152
Head circumference (cm)	0.008	0.383	0.002	0.834	0.034	0.002
Alkaline phosphatase (IU/L)	–0.00004	0.855	0.000	0.407	0.000	0.159
Calcium (mmol/L)	0.075	0.227	0.122	0.040	0.168	0.037
Phosphorus (mmol/L)	0.049	0.157	0.016	0.628	0.065	0.147

**Table 7:** The optimal models for predicting CAS.

	Independent variables^∗^	RMSE	Adjusted R^2^
3.5 MHz	GA	0.26	0.443
	0.008GA – 3.463		
5.0 MHz	GA	0.25	0.405
	0.007GA – 2.705		
7.5 MHz	GA, H, CA	0.33	0.403
	0.006GA+ 0.028H+ 0.172CA – 5.508		

*GA* gestational age, *H* head circumference, *CA* serum calcium, *RMSE* root mean square error of the regression, *R^2^* square of the adjusted correlation coefficient of the regression. ^∗^*P* < 0.001 for GA, *P* = 0.003 for H, *P* = 0.033 for CA.

## Data Availability

The data used to support the findings of this study are available from the corresponding author upon request.
